# Vascular assessment habits in Australian and New Zealand Podiatrists

**DOI:** 10.1186/1757-1146-8-S2-O9

**Published:** 2015-09-22

**Authors:** Peta Craike, Vivienne Chuter

**Affiliations:** 1 Faculty of Health & Medicine, University of Newcastle, Ourimbah, NSW, 2258, Australia

**Keywords:** Vascular assessment, survey, Australian, New Zealand podiatrists

## Background

Podiatrists play a central role in conducting non-invasive vascular assessment in the lower extremity. This involves screening for signs and symptoms of peripheral arterial disease (PAD) and ongoing monitoring of the condition. Podiatric vascular assessment practices in Australia and New Zealand are currently unclear. Determining the clinical habits of Podiatrists is essential in identifying if there is a need for further education or support in performing accurate vascular assessments.

## Methods

A web-based, secure, anonymous questionnaire was conducted of registered Podiatrists in Australia and New Zealand between 1 April and 31 July 2013. The questions examined clinician's regular practices in vascular assessment, along with a short clinical scenario about a fictional patient with complex vascular assessment requirements.

## Results

377 podiatrists participated in the survey. Prompts for vascular assessment, along with barriers and available equipment were examined and the results varied depending on the podiatrists' geographical location, work sector, experience and training. Palpation of pulses was the most frequently reported assessment, followed by temperature gradient and capillary refill time. More podiatrists undertook Doppler assessment (74%) than pressure measurement, with only 34.2% undertaking ankle-brachial indices and 19.4% completing toe-brachial indices. Public podiatrists reported a higher frequency of Doppler use (92%) than private podiatrists (66%). Lack of time was identified as the most frequently reported barrier (66%) in performing vascular assessment, followed by lack of equipment (28%). Time was a much more significant issue in rural podiatrists (77%) compared to their metropolitan colleagues (58%). In New Zealand podiatrists, lack of equipment was much more of an issue, with 43% of podiatrists citing this as a key barrier.

## Conclusions

Large variations exist in vascular assessment methods amongst Australian and New Zealand podiatrists. Some assessments being undertaken are potentially inadequate for accurate screening for PAD. There is a need for continuing education in vascular assessment to address the deficiencies in technique reported by some Podiatrists. Changes in billing structures and health fund rebates for vascular assessment may also assist in addressing the lack of financial incentive for private practitioners.

